# Fast Pyrolysis of Cellulose and the Effect of a Catalyst on Product Distribution

**DOI:** 10.3390/ijerph192416837

**Published:** 2022-12-15

**Authors:** Tanglei Sun, Lu Zhang, Yantao Yang, Yanling Li, Suxia Ren, Lili Dong, Tingzhou Lei

**Affiliations:** 1 Institute of Urban & Rural Mining, Changzhou University, Changzhou 213164, China; 2 Changzhou Key Laboratory of Biomass Green, Safe & High Value Utilization Technology, Changzhou 213164, China

**Keywords:** cellulose, fast pyrolysis, temperature, time, catalyst, product distribution

## Abstract

Fast pyrolysis of microcrystalline cellulose (MC) was carried out by pyrolysis-gas chromatography/mass spectrometry (Py-GC/MS). The effects of temperature, time, and a catalyst on the distribution of the pyrolysis products were analyzed. The reaction temperature and time can significantly affect the types and yields of compounds produced by cellulose pyrolysis. A pyrolysis temperature of 500–600 °C and pyrolysis time of 20 s optimized the yield of volatile liquid in the pyrolysis products of cellulose. In all catalytic experiments, the relative contents of alcohols (1.97%), acids (2.32%), and esters (4.52%) were highest when K_2_SO_4_ was used as a catalyst. HZSM-5 promoted the production of carbohydrates (92.35%) and hydrocarbons (2.20%), while it inhibited the production of aldehydes (0.30%) and ketones (1.80%). MCM-41 had an obvious catalytic effect on cellulose, increasing the contents of aldehydes (41.58%), ketones (24.51%), phenols (1.82%), furans (8.90%), and N-compounds (12.40%) and decreasing those of carbohydrates (5.38%) and alcohols (0%).

## 1. Introduction

As the only carbon-based renewable energy that can be directly converted into liquid fuels, biomass energy has attracted wide attention. Fast pyrolysis is a thermochemical process in which biomass fuels undergo rapid pyrolysis and are condensed in the absence of oxygen. The process is also an economically feasible thermochemical method for transforming biomass into liquid products [1]. Bio-oils (acids, carbohydrates, aldehydes, ketones, hydrocarbons, esters, alcohols, and phenols, etc.) produced by fast pyrolysis can be widely used as fuels and valuable chemicals in many fields [2,3,4], and have gradually emerged as a clean energy that could partially replace traditional fossil fuels. As the most important component of lignocellulosic biomass, cellulose is mainly composed of carbon, hydrogen, and oxygen. Cellulose is a large linear homopolymer with a high degree of polymerization, in which the main component is monomeric glucose that can form fibrils of up to 15,000 glucose units. The internal glucose units in cellulose are connected by a β-1,4-glycosidic bond and have a strong fiber structure [5]. Cellulose is easy to obtain in nature and has a single structure. The structure and chemical characteristics of cellulose obtained from different forms of biomass under similar conditions have good repeatability [6]. To explore the pyrolysis process of biomass, researchers [7,8,9] have attempted to establish a pyrolysis mechanism suitable for biomass involving three components (cellulose, hemicellulose, and lignin), and mainly focused on the generation characteristics of biomass pyrolysis products. Due to the variety of biomass materials and complexity of the pyrolysis process, the pyrolysis mechanism of cellulose should be investigated based on product formation, to provide a reference for clarifying how high-value biomass pyrolysis products are distributed and utilized.

The bio-oil produced by the traditional pyrolysis method has the disadvantages of high moisture and oxygen content, low calorific value and pH, and poor stability [10,11]. The catalytic fast pyrolysis technology is an effective means to produce high-quality bio-oil; it can improve the quality of bio-oil at the source of pyrolysis, which greatly reduces the complexity and difficulty of subsequent upgrading and modification [12,13]. In addition, the pyrolysis temperature and reaction time are important factors affecting the generation of biomass pyrolysis products. Wang et al. investigated pyrolysis temperatures of 500, 550, 650, and 700 °C and found that 600 °C was the most appropriate for the pyrolysis of bamboo [14]. Sun et al. reported that high temperatures (>500 °C) are conducive to the deoxygenation of biomass to generate hydrocarbons, and the bio-oil yield of cornstalk was highest when the pyrolysis time was 10 s [1]. The effect of the duration of the reaction on pyrolysis products was studied by Xu et al., who found that 20 s was beneficial for eucalyptus in terms of the production of aromatic hydrocarbons [15]. To determine the basic parameters needed for the production of high-quality bio-oil by selective pyrolysis, it is necessary to first study the effects of pyrolysis temperature and time on the product distribution of biomass fast pyrolysis. 

Previous studies have shown that, by adjusting the content of inorganic salts in biomass and adding catalysts during the pyrolysis process, the distribution of pyrolysis products can be controlled selectively and the quality of crude bio-oil can be improved [10,16,17]. Leng et al. found that increasing the K^+^ content in biomass promoted dehydration, demethylation, and secondary reactions, which significantly affected the composition of the compounds in bio-oil; the content of lignin-derived phenols, acetone (AT), and glycolaldehyde (GA) was increased, and that of levoglucosan (LG) was greatly reduced [18]. The presence of Zn^2+^ can inhibit the formation of LG, GA, hydroxyacetone (HA), and other small molecular aldehydes and ketones in the pyrolysis products of cellulose, and promote the formation of furan products such as furfural (FF) and 5-methylfurfural (ML), as well as carboxylic acid products such as formic acid (FA) and acetic acid (AA) [15]. As one of the most widely investigated solid acid catalysts for biomass catalytic pyrolysis, molecular sieves are crystalline silicate or silicon aluminate with [SiO_4_] and [AlO_4_]^−^ tetrahedra linked by an oxygen bridge. Molecular sieves have a strong effect on the deoxygenation of bio-oil because of their acidic surface and special pore structure. The special pore structure can stabilize the intermediate products of biomass degradation and prevent the polymerization of intermediate products that form coke; this reduced coke output improves the yield of liquid bio-oil. Moreover, the acidic sites of molecular sieve catalysts can cause dehydration, decarboxylation, dealkylation, cracking, isomerization, oligomerization, and various other reactions; thus, altering the composition and distribution of bio-oil [12,19]. Kim et al. showed that HZSM-5 could promote the generation of aromatic hydrocarbons in the pyrolysis products of cellulose, and the aromatic hydrocarbon yield significantly increased with an increase in the catalyst content [20]. Chi et al. used a modified MCM-41 molecular sieve to study the pyrolysis process of cellulose and found that the proportion of each component in the bio-oil changed significantly during the process [21]. The LG yield decreased significantly after adding a catalyst, while the levoglucosenone yield increased remarkably. The acidity and alkalinity of the metal oxide surface also affected the product distribution in the bio-oil [22]. Zirconium dioxide (ZrO_2_) is an exciting material for various photochemical heterogeneous reactions due to its n-type semiconductor nature; it can also be utilized as a support material for nanoparticle synthesis [23]. Sun et al. reported that ZrO_2_ can effectively convert macromolecular oxygenated compounds into small molecular compounds and increase the bio-oil content in pyrolysis products, promote the generation of aldehydes, alcohols, ketones, and N-compounds, and inhibit the generation of carbohydrates, acids, esters, and phenols [1]. In addition, the introduction of anions (such as SO_4_^2^ and C_1_) on the cellulose surface can improve the yield and quality of bio-oil [17,24]. 

## 2. Experimental Materials and Methods

### 2.1. Experimental Materials

The cellulose used in the experiment was MC (CAS:9004-34-6), which was purchased from Sigma-Aldrich (St. Louis, MO, USA). K_2_SO_4_ (CAS:7778-80-5, AR, 99%), Zinc chloride (ZnCl_2_: CAS:7646-85-7, AR, 98%), and ZrO_2_ (CAS:1314-23-4, 99.99% metals basis, 50 nm) were obtained from Macklin Biochemical Technology Co., Ltd. (Shanghai, China). The zeolite molecular sieves HZSM-5 and MCM-41 were purchased from Nankai University Catalyst Co., Ltd. (Tianjin, China). Proximate and ultimate analyses of MC were conducted based on methods described previously [1,12] and the results are shown in Table 1.

### 2.2. Catalyst Characterization: Nitrogen (N_2_) Adsorption

Nitrogen physical adsorption experiments using HZSM-5 and MCM-41 were performed in an area analyzer (TriStar II 3020; Micromeritics (Shanghai) Instrument Co., Ltd., Shanghai, China) to obtain the specific surface area, pore volume, and pore size. The results are shown in Table 2. Before the test, the samples were degassed under vacuum at 300 °C for 3 h. Subsequently, the samples were precooled to −196 °C and further adsorbed by N_2_ in a liquid nitrogen pool. The specific surface area of samples was calculated and expressed as S_BET_ based on the Brunauer–Emmett–Teller (BET) equation. The total pore volume was characterized by the total absorption at *P/P*_0_ = 0.997. The average pore diameter was calculated as 4 V/A based on the BET equation.

### 2.3. Sample Preparation

Generally, catalysts such as molecular sieves and metal oxides can be mixed with biomass by mechanical mixing. In the study, the MC was mixed with catalysts (HZSM-5, MCM-41, and ZrO_2_) at mass ratios of 3:1, 1:1, and 1:3, respectively, and the mixture was then ground–pressed–reground three times to ensure uniform mixing [12]. The samples were classified according to the raw material, catalyst type, and catalyst content. “MCH31” represented MC mixed with HZSM-5 at a mass ratio of 3:1. The other abbreviations in Table 3 are based on the same naming system. Inorganic salts are usually added to raw materials by soaking. Three grams of MC was immersed in 60 mL K_2_SO_4_ solutions at concentrations of 1, 10, 25, and 50 g/L, respectively. Additionally, 30, 75, 150, and 300 mg of ZnCl_2_ were added to 30 mL deionized water, and 3 g of MC was then added to each solution. The solution was stirred at room temperature for 2 h, and then dried in a drying oven at 105 °C to constant weight. The sample abbreviations were the same as above. MCK1 represents MC immersed in K_2_SO_4_ solution at a concentration of 1 g/L, and MCZn30 represents MC immersed in deionized water supplemented with 30 mg of ZnCl_2_. The other abbreviations in Table 3 are based on the same naming system. After the pretreatment of MC, the concentration of metal ions actually loaded in the raw material was determined according to the method in reference [1]. 

### 2.4. Fast Pyrolysis by Py-GC/MS

**Figure 1:**
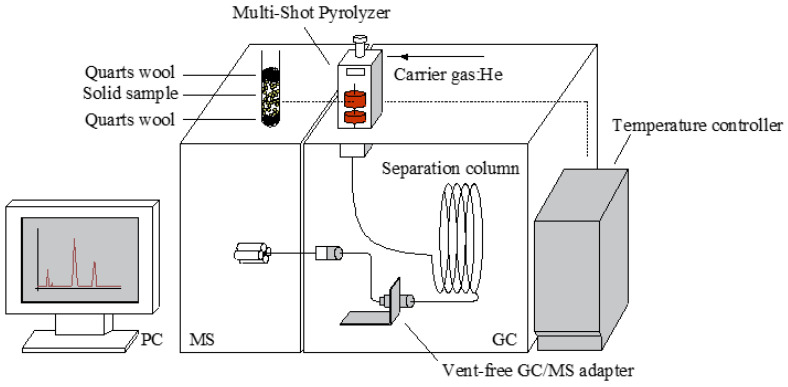
The Py-GC/MS device used in this study.

Reaction temperature and time are decisive factors affecting the distribution of biomass pyrolysis products. Cellulose pyrolysis can be divided into four regions: <300 °C; 300–370 °C; 370–450 °C; and >450 °C [7,25]. Therefore, the MC was pyrolyzed at 285 °C, 345 °C, 445 °C, 500 °C, 600 °C, and 700 °C, and the pyrolysis times were 1, 5, 10, and 20 s. All catalytic fast pyrolysis experiments were conducted for 10 s at 500 °C. Figure 1 shows the Py-GC/MS device used in the study. The pyrolysis process was conducted using a double-click pyrolysis device (EGA/PY-3030D; Frontier Lab, Fukushima, Japan). A 0.1± 0.01 mg sample was placed in a sample cup, and quartz cotton was placed on the upper and lower sides of the sample to prevent solid particles from spilling. The pyrolysis gas was analyzed by GC/MS (QP2010 Ultra; Shimadzu, Kyoto, Japan) with an inlet temperature of 250 °C. An Rtx-5MS capillary column (Restek, Bellefonte, PA, USA; length = 30 m, internal diameter = 0.25 mm, membrane thickness = 0.25 μm) was used for the chromatographic separation. The carrier gas was helium (99.999%) with a flow rate of 1.27 mL/min and split ratio of 100:1. The initial furnace temperature of the gas phase was set to 50 °C for 5 min, and the heating rate was then increased to 260 °C at 10 °C/min for 10 min. The interface temperature and ion source temperature of the GC/MS were set to 280 °C and 230 °C, respectively, and the mass spectrum range was 35–500 m/z. The chromatographic peak was determined by comparison with spectra in the NIST11 spectral library, the F-Search PY-1110E-181 spectral library, and previous data [19,26,27,28]. The chromatographic peak area of each compound in the Py-GC/MS pyrolysis products was proportional to its concentration. Therefore, changes in the yield of a product were determined by comparing the average peak area of each compound obtained under different reaction conditions. The percentage peak area indicates changes in the relative content of the detected products [29,30].

## 3. Results and Discussion

### 3.1. Effect of Reaction Temperature and Time on the Distribution of MC Pyrolysis Products

The cellulose fast-pyrolysis steam was composed of non-condensing gases (CO, CO_2_, CH_4_, H_2_, etc.), volatile compounds, and non-volatile oligomers; condensation of the latter two components formed a liquid bio-oil. The total ion chromatograph of cellulose pyrolysis products contained hundreds of peaks, some of which could not be identified; the unidentifiable peaks are referred to as “others”. To clarify the relationship between cellulose structure and pyrolysis products, cellulose was first pyrolyzed for 10 s at different pyrolysis temperatures without catalyst. 

**Figure 2:**
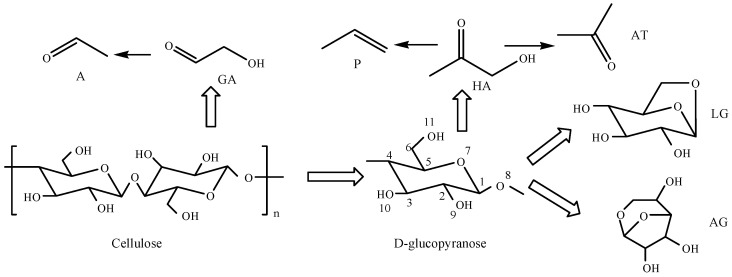
Proposed product formation pathway during cellulose pyrolysis.

As provided in the Supplementary Materials available online, of the 62 main pyrolysis products between 285 °C and 700 °C, only three compounds, LG, 1,6-anhydro-β-D-glucofuranose (AG), and erucamide (E), were detected at all temperatures. The LG yield increased monotonically as the temperature increased from 285 °C to 600 °C, and then began to decrease as the temperature continued to rise to 700 °C. The LG content was very high (>55%) between 345 °C and 700 °C. The AG yield first increased and then decreased with the increase in temperature, and the AG yield and content (6.86%) were highest at 500 °C. When the pyrolysis temperature increased from 285 °C to 500 °C, the E yield increased gradually, and then decreased with a further increase in temperature. The E content was higher at low temperatures (285–345 °C) At the pyrolysis temperature of 285 °C, there were only 10 main pyrolysis products of cellulose, among which E and LG had the highest contents (40.29% and 14.19%, respectively). When the temperature increased to 345 °C, there were only 12 main pyrolysis compounds, among which LG and E still had the highest contents. The LG content significantly increased to 71.22%, and the E content was 12.39%. It was found that LG was generated in large quantities at this temperature. When the pyrolysis temperature was 445 °C, there were 24 main products. The two compounds with the highest contents were the carbohydrates LG (79.13%) and AG (6.50%). Carbohydrates are the most typical products of the cellulose pyrolysis process. The distribution of pyrolysis products at 500–600 °C was similar to that at 445 °C (21 and 25 main products, respectively). The main pyrolysis products were still LG and AG, accounting for 77.06% and 6.86%, and 78.14% and 6.11%, of all products at each temperature, respectively. When the pyrolysis temperature increased to 700 °C, there were 33 main pyrolysis products. The five compounds with the highest contents were LG (57.69%), AT (6.11%), propylene (P) (5.90%), acetaldehyde (A) (5.69%), and AG (4.64%), of which AT and P were only produced at 700 °C. Acetaldehyde was produced between 345 °C and 700 °C, and its yield increased with temperature. The content of small-molecule straight-chain products increased significantly at high temperatures. From the perspective of product formation and evolution, the mechanism of cellulose pyrolysis can be summarized as shown in Figure 2. At the initial stage of cellulose pyrolysis, carbohydrates with a low degree of polymerization were first generated by a depolymerization reaction, and then further decomposed into glucopyranose units through breakage of the β-1,4-glycosidic bond. The formation of LG occurs due to a condensation reaction between the hydroxyl group on C_6_ and oxygen free radical on C_1_. When C_1_ and C_4_ are dehydrated, the pyrane-type glucose unit will generate AG, which competes with the formation of LG; the generation of AT, P, and A also competes with the formation of AG and LG. The formation of A occurs due to the dehydroxylation of GA, while the generation of AT and P is related to the dehydroxylation and polycondensation of HA [30,31,32].

**Figure 3:**
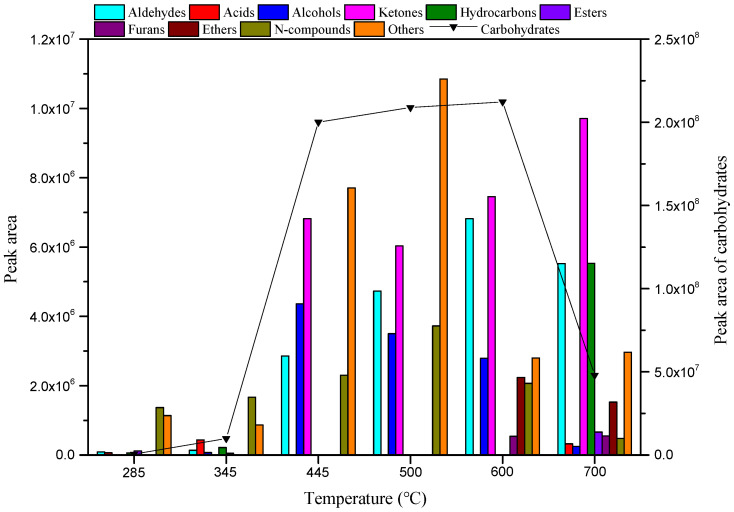
Yields of various compounds in bio-oil derived from MC pyrolysis at different temperatures for 10 s.

**Figure 4:**
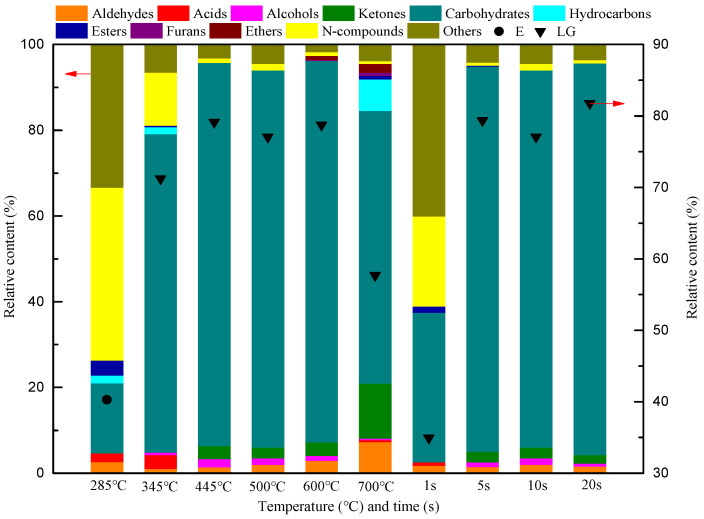
Distribution of MC pyrolysis products at different temperatures and reaction times.

The yield and distribution of various compounds in cellulose pyrolysis bio-oil at different temperatures are shown in Figure 3 and Figure 4. Figure 3 shows that the pyrolysis of cellulose produced a variety of compounds, including aldehydes, acids, alcohols, ketones, carbohydrates, hydrocarbons, esters, furans, ethers, and N-compounds. According to the sum of the peak areas of the various compounds in Figure 3, the total peak areas of each temperature between 285 °C and 700 °C were 3.41 × 10^6^, 1.34 × 10^7^, 2.24 × 10^8^, 2.38 × 10^8^, 2.37 × 10^8^, and 7.55 × 10^7^, respectively. At the pyrolysis temperature of 500–600 °C the yield of volatile liquid was substantially improved, and the yield of many compounds changed significantly with the change of pyrolysis temperature, indicating that the reaction temperature significantly affected the type and yield of compounds produced by cellulose pyrolysis.

Figure 3 shows that the aldehyde yield gradually increased as the temperature increased from 285 °C to 600 °C, and then began to decrease as the temperature increased from 600 °C to 700 °C. There was no obvious pattern to the formation of acid compounds in the experimental temperature range; the highest yield occurred at 345 °C and there was no production between 445 °C and 600 °C. Alcohols were not produced at the lowest temperature (285 °C), and their formation displayed a trend of first increasing and then decreasing between 345 °C and 700 °C; the yield reached a maximum at 445 °C. Ketones were not produced at low temperatures (285–345 °C), and the highest production was achieved at 700 °C. Below 600 °C, the carbohydrate yield increased monotonically with the increase in temperature. When the temperature was >600 °C, the carbohydrate yield decreased sharply with the increasing temperature. Figure 4 shows the distribution of various compounds in bio-oil from MC pyrolysis at different temperatures and times. At 345 °C, 445 °C, 500 °C, 600 °C, and 700 °C, the carbohydrates were the most important pyrolysis products, with relative contents of 74.27%, 89.41%, 87.95%, 88.99%, and 63.60%, respectively. The LG content was highest at 345 °C, 445 °C, 500 °C, 600 °C, and 700 °C, accounting for 71.22%, 79.13%, 77.06%, 78.74%, and 57.69% of the total products, respectively. Hydrocarbons were generated only at lower temperatures (285–345 °C) and at the highest temperature (700 °C), with the highest yield achieved at 700 °C. A small amount of the hydrocarbons produced at low temperatures were alkanes with multiple carbon atoms, while at high temperatures they were mostly olefins and aromatic hydrocarbons with simple structures. This shows that higher temperatures resulted in more complete cellulose decomposition, and the product type was simpler. In the experimental range, the ester yield first decreased and then increased with the increase in temperature, and reached a maximum at 700 °C. Furans and ethers were generated only at 600–700 °C, and their highest yields occurred at 600 °C and 700 °C, respectively. As the temperature increased from 285 °C to 500 °C, the yield of N-compounds increased gradually, and then began to decrease as the temperature increased from 500 °C to 700 °C. N-compounds were the most important pyrolysis products at 285 °C, with a content of 40.29%. At 285 °C, E was the most abundant compound, accounting for 40.29% of the pyrolysis products (Figure 4). 

Reaction time is another important factor affecting the distribution of cellulose pyrolysis products. As shown in the Supplementary Materials available online, among the 27 major pyrolysis products that formed between 1 and 20 s, only FF, LG, and E were detected at all pyrolysis times. With the increase in pyrolysis time, the FF yield increased gradually, but its relative content was low (<1%) over the entire experimental range. However, FF was the main product during hemicellulose pyrolysis. Considering the closely connected structure of the three biomass components, it was inferred that the generation of FF at this time was mainly related to hemicellulose pyrolysis, with hemicellulose not being completely removed from cellulose. Similar to FF, the LG yield increased monotonically with the increase in pyrolysis time from 1 to 20 s; the content was relatively high between 1 and 20 s, especially at 5–20 s (about 80%). The E yield first increased and then decreased with the increase in pyrolysis time, reaching its highest level at 10 s. The E content was very high when the pyrolysis time was short (1 s), but was low between 5 and 20 s (about 1%). During pyrolysis for 1 s, six main compounds were produced, among which LG and E had the highest yields; they accounted for 34.93% and 20.99% of all products, respectively. As the pyrolysis time increased to 5–20 s, the number of different pyrolysis products reached 21, and most compounds were detected at all times; this indicated that the influence of reaction time on the types of compounds generated during pyrolysis was much less than that of temperature. The two compounds with the highest yield from 5 s to 20 s were LG and AG. At 5, 10, and 20 s, the relative contents of the two compounds were 79.35% and 6.98%, 77.06% and 6.86%, and 81.75% and 6.03%, respectively. The production of AG only occurred between 5 and 20 s, and its yield first increased and then decreased with the increase in pyrolysis time; the highest yield occurred at 10 s.

**Figure 5:**
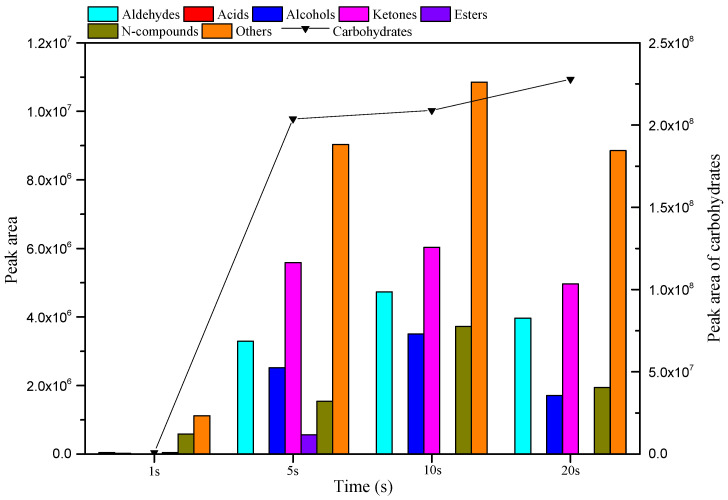
Yields of various compounds in bio-oil obtained from MC pyrolysis at 500 °C with different reaction times.

The content and yield distribution of various compounds in the pyrolysis bio-oil when MC was pyrolyzed for different times at 500 °C are shown in Figure 4 and Figure 5. According to the sum of the peak areas of various compounds shown in Figure 5, it can be concluded that the total peak areas at pyrolysis times of 1, 5, 10, and 20 s were 2.79 × 10^6^, 2.26 × 10^8^, 2.38 × 10^8^, and 2.49 × 10^8^, respectively. It can be seen that the pyrolysis time of 20 s improved the yield of volatile liquid. As shown in Figure 5, the yield of all compounds significantly increased with pyrolysis time from 1 to 5 s, while the yield of all compounds changed little between 5 and 20 s. As the pyrolysis time increased from 1 to 10 s, the yields of aldehydes, alcohols, ketones, and N-compounds displayed an increasing trend. As the pyrolysis time continued to increase from 10 to 20 s, the yields of these compounds began to decrease. Acids were only produced in trace amounts at a pyrolysis time of 1 s, and esters were only produced during a short pyrolysis time (1–5 s), with the highest yield occurring at 5 s. The yield of carbohydrates increased with the increasing pyrolysis time, and reached a maximum at 20 s. Carbohydrates were the main product of cellulose pyrolysis from 1 to 20 s, with relative contents of 34.93%, 89.84%, 87.95%, and 91.34% for 1, 5, 10, and 20 s, respectively. The compound with the highest relative content after pyrolysis of 1–20 s was LG, accounting for 34.93%, 79.35%, 77.06%, and 81.75 of all products at 1, 5, 10, and 20 s, respectively (Figure 4).

### 3.2. Effect of Catalyst Type and Amount on the Pyrolysis Product Distribution of MC

**Figure 6:**
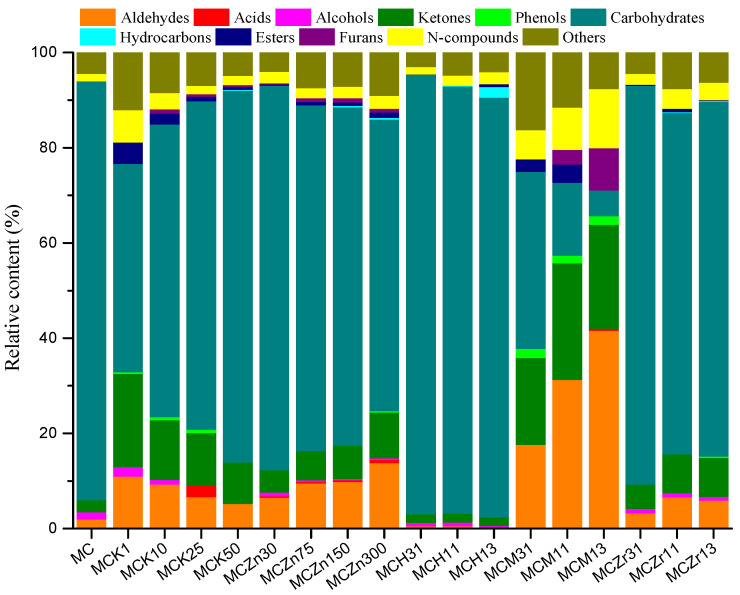
Distribution of bio-oil products from the catalytic pyrolysis of MC.

**Figure 7:**
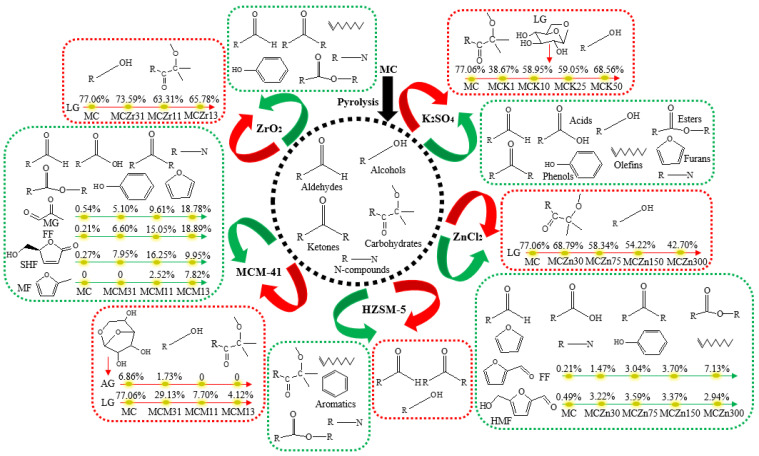
Effects of five catalysts on the distribution of MC pyrolysis products (green and red represent promotional and inhibitory effects, respectively) (LG, Levoglucosan; MG, Methylglyoxal; FF, Furfural; SHF, (S)-5-Hydroxymethyl-2[5 H]-furanone; MF, 2-Methylfuran; AG, 1,6-Anhydro-β-D-glucofuranose; HMF, 5-Hydroxymethylfurfural).

Five different catalysts, K_2_SO_4_, ZnCl_2_, HZSM-5, MCM-41, and ZrO_2_ were used to conduct the catalytic fast pyrolysis of MC; the results are shown in Figure 6 and Figure 7. As shown in Figure 6, introducing K_2_SO_4_ into MC significantly increased the aldehyde content from 2.00% (MC) to 10.98% (MCK1), 9.33% (MCK10), 6.65% (MCK25), and 5.21% (MCK50), and the ketone content from 2.54% (MC) to 19.55% (MCK1), 12.51% (MCK10), 11.09% (MCK25), and 8.75% (MCK50). The potassium ion significantly promoted these two kinds of compounds when the amount of catalyst was low, and the promotional effect decreased with the increase in catalyst content. The increase in contents of these two compounds was not reflected in the increase in content of one or more compounds, but the introduction of K^+^ increased the number of different types of these two compounds, thereby improving their relative contents. The five catalysts had no obvious effect on the acid production of MC, and the acid content reached a maximum of only 2.32% at MCK25. In the presence of K_2_SO_4_, small amounts of catalyst (MCK1) slightly promoted the formation of alcohols, while large amounts of catalysts (MCK10, MCK25, and MCK50) inhibited the production of alcohols until the content was 0%. Small amounts of K_2_SO_4_ (MCK1, MCK10, and MCK25) promoted the production of some phenolic compounds, while hydrocarbons (olefins) were produced only in trace amounts with the addition of MCK50. Similar to the aldehydes and ketones, K^+^ had a promotional effect on esters and N-compounds when small amounts were added, but this effect decreased with increasing catalyst content. Large amounts of catalysts (MCK10, MCK25, and MCK50) had a small promotional effect on furan formation. Using K_2_SO_4_ as a catalyst, the carbohydrate yield was significantly reduced, and its content decreased from 87.95% to 43.76% (MCK1), 61.49% (MCK10), 68.98% (MCK25), and 78.05% (MCK50). The inhibitory effect on carbohydrates also decreased with increasing catalyst content. The decrease in production of carbohydrates was mainly reflected in the decrease in LG content, from 77.06% to 38.67% (MCK1), 58.95% (MCK10), 59.05% (MCK25), and 68.56% (MCK50) (Figure 7). In summary, the addition of K_2_SO_4_ had opposing effects on the formation of compounds. This may be because K^+^ and SO_4_^2^ have opposite effects on the distribution and composition of pyrolysis products. For example, some studies have reported that the presence of K^+^ reduces the formation of LG, while the introduction of SO_4_^2^ can improve the selectivity of LG and increase its production [1,33]. In addition, in all catalytic experiments the highest contents of alcohols (1.97%) and esters (4.52%) were obtained using MCK1.

Figure 6 shows that using ZnCl_2_ as a catalyst increased the aldehyde content. The aldehyde content increased with the increase in ZnCl_2_, from 2.00% (MC) to 6.58% (MCZn30), 9.55% (MCZn75), 9.86% (MCZn150), and 13.79% (MCZn300). The increase in aldehyde content was mainly due to the increase in FF and 5-hydroxymethylfurfural (HMF), from 0.21% (MC) to 1.47% (MCZn30), 3.04% (MCZn75), 3.70% (MCZn150), 7.13% (MCZn300), and 0.49% (MC) to 3.22% (MCZn30), 3.59% (MCZn75), 3.37% (MCZn150), and 2.94% (MCZn300), respectively (Figure 7). In the presence of ZnCl_2_, MC promotion produced acids, esters, and N-compounds, although the yields were low. The alcohol yield was slightly inhibited under the catalysis of ZnCl_2_, while the furan content was slightly increased. Using ZnCl_2_ as a catalyst, the ketone content increased from 2.54% (MC) to 9.62% (MCZn300). Phenols and hydrocarbons were produced only when the ZnCl_2_ content was high, although the yields were very low (< 0.5%). In the presence of ZnCl_2_, the production of carbohydrates was significantly inhibited, and the inhibitory effect increased with the increased amount of ZnCl_2_ added. The carbohydrate content decreased from 87.95% (MC) to 80.76% (MCZn30), 72.64% (MCZn75), 71.09% (MCZn150), and 61.24% (MCZn300). The decrease in carbohydrate content was mainly due to the decreasing LG content, from 77.06% to 68.79% (MCZn30), 58.34% (MCZn75), 54.22% (MCZn150), and 42.70% (MCZn300) (Figure 7).

Figure 6 shows that using HZSM-5 as a catalyst slightly reduced the contents of aldehydes, alcohols, and ketones, although changes in the amount of catalyst added had no obvious effect on these three compounds. HZSM-5 had no promotional effect on the acid, phenol, and furan production from MC, with the yield of these three compounds always being 0%. HZSM-5 had a slight promotional effect on the relative content of N-compounds. Large amounts of HZSM-5 (MCH13) promoted the formation of esters, although the relative content was only 0.56%. Among the catalytic experiments, the maximum hydrocarbon content (2.20%) occurred with the addition of MCH13, with all compounds being olefins or aromatics. HZSM-5 was the only catalyst that increased the carbohydrate content, from 87.95% (MC) to 92.35% (MCH31), 89.73% (MCH11), and 88.10% (MCH13). In the catalytic experiments using MC, the minimum contents of aldehydes (0.30%) and ketones (1.80%) were obtained with the addition of MCH13 and MCH31, respectively. 

Using MCM-41 as a catalyst resulted in an increase in relative aldehyde content, from 2.00% (MC) to 17.63% (MCM31), 31.26% (MCM11), and 41.58% (MCM13) (Figure 6). The increasing aldehyde content was mainly due to the increase in methylglyoxal (MG) and FF. The MG content increased from 0.54% (MC) to 5.10% (MCM31), 9.61% (MCM11), and 18.78% (MCM13), while the FF content increased from 0.21% (MC) to 6.60% (MCM31), 15.05% (MCM11), and 18.89% (MCM31) (Figure 7). The formation of MG can be explained by the ring-opening of glucopyranose to form D-glucose, and the subsequent breakage of the C_-2_-C_-3_ bond in retro-aldol mode to form GA and erythrose (ET). In addition, ET could be formed by the cleavage of pyrane-type L-glucan through C_-6_-O-C_-1_ and C_-2_-C_-3_. The decarbonylation and dehydration reactions of ET produce CO, H_2_O, and HA, while the dehydration and dehydroxyl-methyl reactions produce formaldehyde and MG. The formation of FF mainly occurs due to the ring-opening of glucopyranose, which generates D-glucose, and then through the formation of the furan ring, carbon bond breakage, and dehydration to generate FF, or from the secondary decomposition of HMF [34,35]. 

The formation of acids from MC was only slightly promoted by MCM-41. The acid content was only 0.44% in the presence of MCM13, while the alcohol content was 0% in the presence of MCM-41. The relative ketone content increased significantly with the addition of MCM-41, from 2.54% (MC) to 18.27% (MCM31), 24.51% (MCM11), and 21.83% (MCM13) (Figure 6). The increase in ketone content was mainly due to the formation of (S)-5-hydroxymethyl-2[5H]-furanone (SHF), which increased from 0.27% (MC) to 7.95% (MCM31), 16.25% (MCM11), and 9.95% (MCM13) (Figure 7). The presence of MCM-41 promoted the pyrolysis of MC to produce a small amount of phenols. At the same time, the addition of MCM-41 greatly reduced the carbohydrate content, from 87.95% (MC) to 37.20% (MCM31), 15.30% (MCM11), and 5.38% (MCM13). The decrease in carbohydrate content was mainly due to the decrease in LG and AG contents from 77.06% to 29.13% (MCM31), 7.70% (MCM11), 4.12% (MCM13), and from 6.86% to 1.73% (MCM31), 0 % (MCM11), and 0% (MCM13), respectively (Figure 7). MCM-41 had no effect on the formation of hydrocarbons from MC, which had a relative content of 0% before and after adding the catalyst. The low amounts of MCM-41 (MCM31, MCM11) promoted the production of small amounts of esters, while the high amount of MCM-41 significantly promoted the production of furans. The furan content increased from 0% (MC) to 8.90% (MCM13). The change in furan content was mainly due to the increase in the 2-methylfuran (MF) content from 0% (MC) to 7.82% (MCM13) (Figure 7). Among the five different catalysts, only MCM-41 promoted the production of furans. The production of N-compounds was promoted by MCM-41, with the content increasing from 1.56% (MC) to 6.16% (MCM31), 8.90% (MCM11), and 12.40% (MCM13) (Figure 6). Among all catalytic experiments, MCM13 obtained the highest contents of aldehydes, ketones, phenols, furans, and N-compounds, and the lowest content of carbohydrates, indicating that the addition of a large amount of MCM-41 strongly promoted the production of aldehydes, ketones, phenols, furans, and N-compounds and had a strong inhibitory effect on the generation of carbohydrates.

The addition of ZrO_2_ had a promotional effect on the pyrolysis of MC, which produced aldehydes; their relative content increased from 2.00% (MC) to 3.26% (MCZr31), 6.60% (MCZr11), and 5.91% (MCZr13) (Figure 6). However, it had no effect on the production of acids or furans from MC, and the yields of these two compounds were always 0%. Using ZrO_2_ as a catalyst slightly inhibited the formation of alcohols, and the inhibitory effect had no clear relationship with the amount of catalyst added. The ketone content increased in the presence of ZrO_2_, and with the increase in the amount of catalyst added. Phenols were produced only when the amount of ZrO_2_ added was high (MCZr13), but the relative content was only 0.31%. Using ZrO_2_ as a catalyst promoted an increase in the contents of hydrocarbons, esters, and N-compounds, but changes in the catalyst content had little effect on the production of these three compounds. The presence of ZrO_2_ also significantly inhibited the production of carbohydrates, with their relative content decreasing from 87.95% (MC) to 83.58% (MCZr31), 71.66% (MCZr11), and 74.46% (MCZr13) (Figure 6). The decrease in carbohydrate content was mainly due to the decrease in LG content, from 77.06% to 73.59% (MCZr31), 63.31% (MCZr11), and 65.78% (MCZr13) (Figure 7).

## 4. Conclusions

Reaction temperature and time can significantly affect the types and yields of compounds produced by cellulose pyrolysis. Under a pyrolysis temperature of 500–600 °C and pyrolysis time of 20 s, the yield of volatile liquid in cellulose pyrolysis products was improved. In all the catalytic experiments, the contents of alcohols (1.97%), acids (2.32%), and esters (4.52%) were highest when K_2_SO_4_ was used as a catalyst. HZSM-5 promoted the production of carbohydrates (92.35%) and hydrocarbons (2.20%), and inhibited the formation of aldehydes (0.30%) and ketones (1.80%). MCM-41 had a significant catalytic effect on cellulose; its addition increased the relative contents of aldehydes (41.58%), ketones (24.51%), phenols (1.82%), furans (8.90%), and N-compounds (12.40%), and reduced those of carbohydrates (5.38%) and alcohols (0%).

## Figures and Tables

**Table 1:** Chemical characteristics of the MC.

Sample	Proximate Analysis (wt.%)				Ultimate Analysis (wt.%)				
	Moisture	Volatile	Fixed Carbon	Ash	C	H	O	N	S
MC	6.58	87.32	5.58	0.52	44.76	6.01	48.15	0.51	0.05

**Table 2:** Properties of the catalysts.

Sample	Si/Al	Crystallinity (%)	Crystal Size (μm)	S_BET_ (m^2^/g)	Pore Volume (mL/g)	Avg. Pore Diameter (nm)	Adsorption n-Hexane (mg/g)	Na_2_O (% m/m)	LOI (% m/m)
HZSM-5	46	95	0.5–1	350	0.110	0.253	-	0.08	≤10
MCM-41	25	91	-	750	0.728	3.531	90	0.79	13

Notes: loss on ignition (LOI).

**Table 3:** Sample abbreviations and metal ion content.

Sample	Abbreviation	The K^+^ or Zn^2+^ Content
MC:HZSM-5 = 3:1	MCH31	-
MC:HZSM-5 = 1:1	MCH11	-
MC:HZSM-5 = 1:3	MCH13	-
MC:MCM-41 = 3:1	MCM31	-
MC:MCM-41 = 1:1	MCM11	-
MC:MCM-41 = 1:3	MCM13	-
MC:ZrO_2_ = 3:1	MCZr31	-
MC:ZrO_2_ = 1:1	MCZr11	-
MC:ZrO_2_ = 1:3	MCZr13	-
MC + K_2_SO_4_ 1 g/L	MCK1	0.17%
MC + K_2_SO_4_ 10 g/L	MCK10	0.39%
MC + K_2_SO_4_ 25 g/L	MCK25	0.65%
MC + K_2_SO_4_ 50 g/L	MCK50	1.05%
MC + ZnCl_2_ 30 mg	MCZn30	0.48%
MC + ZnCl_2_ 75 mg	MCZn75	0.78%
MC + ZnCl_2_ 150 mg	MCZn150	1.07%
MC + ZnCl_2_ 300 mg	MCZn300	1.25%

## Supplementary Materials

The following supporting information can be downloaded at: https://www.mdpi.com/article/10.3390/ijerph192416837/s1, Table S1: The product distributions from the non-catalytic pyrolysis of MC at different temperatures; Table S2: The product distributions from the non-catalytic pyrolysis of MC at different time.
