# Supplementary material for: Fast Pyrolysis of Cellulose and the Effect of a Catalyst on Product Distribution
Source: Int J Environ Res Public Health. 2022 Dec 15;19(24):16837. doi: 10.3390/ijerph192416837 (PMC9779704; doi:10.3390/ijerph192416837)
Supplement: Supplementary file 1 [file ijerph-19-16837-s001.zip › ijerph-2066728-supplementary.pdf]

Table S1. The product distributions from the non-catalytic pyrolysis of MC at different temperatures.

Category	Compounds	285℃		345℃		445℃		500℃		600℃		700℃	
		Peak area	Peak area/%	Peak area	Peak area/%	Peak area	Peak area/%	Peak area	Peak area/%	Peak area	Peak area/%	Peak area	Peak area/%
Aldehydes	Glyoxal	-	-	-	-	-	-	4.93×10^{5}	0.21	-	-	-	-
	Methylglyoxal	-	-	-	-	8.64×10^{5}	0.39	1.27×10^{6}	0.54	3.34×10^{6}	1.41	-	-
	Succindialdehyde	-	-	-	-	-	-	-	-	-	-	2.73×10^{5}	0.36
	Furfural	-	-	-	-	4.92×10^{5}	0.22	5.05×10^{5}	0.21	5.86×10^{5}	0.25	2.54×10^{5}	0.34
	Acetaldehyde	-	-	-	-	5.51×10^{5}	0.25	7.70×10^{5}	0.32	2.58×10^{6}	1.09	4.30×10^{6}	5.69
	5-Hydroxymethylfurfural	-	-	-	-	9.53×10^{5}	0.43	1.15×10^{6}	0.49	3.16×10^{5}	0.13	-	-
	Undecanal	-	-	-	-	-	-	5.45×10^{5}	0.23	-	-	-	-
	Nonanal	8.94×10^{4}	2.63	1.37×10^{5}	1.02	1.89×10^{5}	0.08	-	-	-	-	-	-
	Pentanal, 2,4-dimethyl-	-	-	-	-	-	-	-	-	-	-	3.10×10^{5}	0.41
	Crotonaldehyde	-	-	-	-	-	-	-	-	-	-	2.75×10^{5}	0.36
	2-Furaldehyde, 5-methyl-	-	-	-	-	-	-	-	-	-	-	1.11×10^{5}	0.15
Acids	Acrylic acid	-	-	-	-	-	-	-	-	-	-	3.24×10^{5}	0.43
	Palmitic acid	6.80×10^{4}	2.00	2.33×10^{5}	1.74	-	-	-	-	-	-	-	-
	Nonanoic acid	-	-	1.04×10^{5}	0.77	-	-	-	-	-	-	-	-
	Oleic Acid	-	-	4.63×10^{4}	0.34	-	-	-	-	-	-	-	-
	Stearic acid	-	-	5.53×10^{4}	0.41	-	-	-	-	-	-	-	-
Alcohols	1,3-Propanediol, 2-ethyl-2-(hydroxymethyl)-	-	-	-	-	2.66×10^{6}	1.18	2.72×10^{6}	1.14	1.71×10^{6}	0.72	-	-
	Isosorbide	-	-	-	-	-	-	-	-	5.26×10^{5}	0.22	2.49×10^{5}	0.33
	1-Dodecanol	-	-	-	-	-	-	7.82×10^{5}	0.33	-	-	-	-
	2-Furanmethanol	-	-	7.46×10^{4}	0.56	5.43×10^{5}	0.24	-	-	-	-	-	-
	1,3-Diamino-2-propanol	-	-	-	-	1.16×10^{6}	0.52	-	-	-	-	-	-
	cis-1,2-Cyclohexanediol	-	-	-	-	-	-	-	-	5.57×10^{5}	0.24	-	-
Ketones	Acetone	-	-	-	-	-	-	-	-	-	-	4.61×10^{6}	6.11
	2,3-Butanedione	-	-	-	-	-	-	-	-	1.88×10^{6}	0.79	1.05×10^{6}	1.39
	Hydroxyacetone	-	-	-	-	2.18×10^{5}	0.10	-	-	8.89×10^{5}	0.38	1.92×10^{6}	2.55
	Acetoin	-	-	-	-	-	-	-	-	-	-	2.89×10^{5}	0.38
	1,4-Pentadien-3-one	-	-	-	-	-	-	-	-	5.32×10^{5}	0.22	-	-
	(S)-5-Hydroxymethyl-2[5H]-furanone	-	-	-	-	3.80×10^{5}	0.17	6.52×10^{5}	0.27	7.03×10^{5}	0.30	3.40×10^{5}	0.45
	2-Cyclopenten-1-one	-	-	-	-	-	-	-	-	-	-	1.84×10^{5}	0.24

	2,5-Dimethyl-4-hydroxy-3(2H)-furanone	-	-	-	-	-	-	3.30×10^{5}	0.14	2.86×10^{5}	0.12	-	-
	2-Hydroxy-6,8-dioxabicyclo[3.2.1]octan-4-one	-	-	-	-	5.71×10^{6}	2.55	5.05×10^{6}	2.13	2.53×10^{6}	1.07	-	-
	5-Methylfuran-2(3H)-one	-	-	-	-	1.68×10^{5}	0.08	-	-	6.36×10^{5}	0.27	7.43×10^{5}	0.98
	4H-Pyran-4-one, 2,3-dihydro-3,5-dihydroxy-6-methyl-	-	-	-	-	1.73×10^{5}	0.08	-	-	-	-	-	-
	4H-Pyran-4-one, 3,5-dihydroxy-2-methyl-	-	-	-	-	1.67×10^{5}	0.07	-	-	-	-	-	-
	2H-Pyran-2-one	-	-	-	-	-	-	-	-	-	-	2.22×10^{5}	0.30
	4H-Pyran-4-one	-	-	-	-	-	-	-	-	-	-	2.11×10^{5}	0.28
	2-Cyclopenten-1-one, 2-hydroxy-3-methyl-	-	-	-	-	-	-	-	-	-	-	1.42×10^{5}	0.19
Carbohydrates	DL-Xylose	-	-	-	-	1.88×10^{5}	0.08	4.38×10^{5}	0.18	-	-	-	-
	3,6-Dianhydro-alpha-glucopyranose	-	-	-	-	5.16×10^{5}	0.23	1.14×10^{6}	0.48	1.74×10^{6}	0.74	6.14×10^{5}	0.81
	1,4:3,6-Dianhydro-.alpha.-d-glucopyranose	-	-	-	-	6.14×10^{5}	0.27	6.06×10^{5}	0.25	4.93×10^{5}	0.21	1.96×10^{5}	0.26
	D-Allose	-	-	-	-	3.73×10^{6}	1.66	3.04×10^{6}	1.28	2.61×10^{6}	1.10	-	-
	Levoglucosan	4.83×10^{5}	14.19	9.56×10^{6}	71.22	1.77×10^{8}	79.13	1.83×10^{8}	77.06	1.88×10^{8}	78.74	4.35×10^{7}	57.69
	.alpha.-D-Glucopyranose, 4-O-.beta.-D-galactopyranosyl-	-	-	-	-	3.45×10^{6}	1.54	4.39×10^{6}	1.84	4.96×10^{6}	2.09	1.54×10^{5}	0.20
	1,6-Anhydro-.beta.-D-glucofuranose	7.46×10^{4}	2.19	4.09×10^{5}	3.05	1.46×10^{7}	6.50	1.63×10^{7}	6.86	1.45×10^{7}	6.11	3.50×10^{6}	4.64
Hydrocarbons	Propene	-	-	-	-	-	-	-	-	-	-	4.46×10^{6}	5.90
	1,3-Cyclohexadiene	-	-	-	-	-	-	-	-	-	-	2.77×10^{5}	0.37
	Toluene	-	-	-	-	-	-	-	-	-	-	5.85×10^{5}	0.77
	Heptane, 3-methylene-	-	-	-	-	-	-	-	-	-	-	1.08×10^{5}	0.14
	Hexadecane	3.57×10^{4}	1.05	1.14×10^{5}	0.85	-	-	-	-	-	-	-	-
	Dodecane, 4,6-dimethyl-	2.58×10^{4}	0.76	-	-	-	-	-	-	-	-	-	-
	Eicosane	-	-	1.01×10^{5}	0.75	-	-	-	-	-	-	-	-
	1-Decene, 2,4-dimethyl-	-	-	-	-	-	-	-	-	-	-	1.05×10^{5}	0.14
Esters	Propanoic acid, 2-hydroxy-, methyl ester, (.+/-.)-	-	-	-	-	-	-	-	-	-	-	6.64×10^{5}	0.88

	1,2-Benzenedicarboxylic acid, bis(2-methylpropyl) ester	3.39×10^4	1.00	-	-	-	-	-	-	-	-	-	-
	Dibutyl phthalate	3.41×10^4	1.00	5.22×10^4	0.39	-	-	-	-	-	-	-	-
	Bis(2-ethylhexyl) phthalate	5.16×10^4	1.52	-	-	-	-	-	-	-	-	-	-
Furans	Furan, 2-methyl-	-	-	-	-	-	-	-	-	5.42×10^5	0.23	1.67×10^5	0.22
	Furan, 2,5-dihydro-	-	-	-	-	-	-	-	-	-	-	3.86×10^5	0.51
Ethers	Ethyl vinyl ether	-	-	-	-	-	-	-	-	2.24×10^6	0.95	1.53×10^6	2.03
N-compounds	2-Propenamide, N-(aminocarbonyl)-	-	-	-	-	1.79×10^5	0.08	3.39×10^5	0.14	3.21×10^5	0.14	-	-
	Oxazolidine, 2,2-diethyl-3-methyl-	-	-	-	-	2.05×10^5	0.09	3.17×10^5	0.13	3.07×10^5	0.13	-	-
	Erucamide	1.37×10^6	40.29	1.67×10^6	12.39	1.92×10^6	0.86	3.07×10^6	1.29	1.44×10^6	0.61	4.82×10^5	0.64

Table S2. The product distributions from the non-catalytic pyrolysis of MC at different time.

Category	Compounds	1s		5s		10s		20s	
		Peak area	Peak area/%	Peak area	Peak area/%	Peak area	Peak area/%	Peak area	Peak area/%
Aldehydes	Glyoxal	-	-	4.14×10^{5}	0.18	4.93×10^{5}	0.21	4.79×10^{5}	0.19
	Methylglyoxal	-	-	9.88×10^{5}	0.44	1.27×10^{6}	0.54	1.25×10^{6}	0.50
	Furfural	2.17×10^{4}	0.78	4.18×10^{5}	0.18	5.05×10^{5}	0.21	5.29×10^{5}	0.21
	Acetaldehyde	-	-	5.16×10^{5}	0.23	7.70×10^{5}	0.32	7.48×10^{5}	0.30
	5-Hydroxymethylfurfural	-	-	9.03×10^{5}	0.40	1.15×10^{6}	0.49	9.58×10^{5}	0.38
	Undecanal	-	-	-	-	5.45×10^{5}	0.23	-	-
	Nonanal	2.62×10^{4}	0.94	-	-	-	-	-	-
Acids	Palmitic acid	2.25×10^{4}	0.80	-	-	-	-	-	-
Alcohols	1,3-Propanediol, 2-ethyl-2-(hydroxymethyl)-	-	-	2.52×10^{6}	1.11	2.72×10^{6}	1.14	1.71×10^{6}	0.69
	1-Dodecanol	-	-	-	-	7.82×10^{5}	0.33	-	-
Ketones	Hydroxyacetone	-	-	-	-	-	-	2.43×10^{5}	0.10
	1,4-Pentadien-3-one	-	-	-	-	-	-	2.83×10^{5}	0.11
	(S)-5-Hydroxymethyl-2[5H]-furanone	-	-	3.99×10^{5}	0.18	6.52×10^{5}	0.27	6.54×10^{5}	0.26
	Cyclopentanone, 2-methyl-	-	-	-	-	-	-	2.21×10^{5}	0.09
	2,5-Dimethyl-4-hydroxy-3(2H)-furanone	-	-	1.97×10^{5}	0.09	3.30×10^{5}	0.14	2.32×10^{5}	0.09
	2-Hydroxy-6,8-dioxabicyclo[3.2.1]octan-4-one	-	-	4.99×10^{6}	2.20	5.05×10^{6}	2.13	3.33×10^{6}	1.33
Carbohydrates	DL-Xylose	-	-	4.42×10^{5}	0.20	4.38×10^{5}	0.18	3.06×10^{5}	0.12
	3,6-Dianhydro-alpha-glucopyranose	-	-	1.39×10^{6}	0.61	1.14×10^{6}	0.48	7.44×10^{5}	0.30
	1,4:3,6-Dianhydro-.alpha.-d-glucopyranose	-	-	5.58×10^{5}	0.25	6.06×10^{5}	0.25	4.40×10^{5}	0.18
	D-Allose	-	-	1.65×10^{6}	0.73	3.04×10^{6}	1.28	3.76×10^{6}	1.51
	Levoglucosan	9.76×10^{5}	34.93	1.80×10^{8}	79.35	1.83×10^{8}	77.06	2.04×10^{8}	81.75
	.alpha.-D-Glucopyranose, 4-O-.beta.-D-galactopyranosyl-	-	-	3.90×10^{6}	1.72	4.39×10^{6}	1.84	3.61×10^{6}	1.45
	1,6-Anhydro-.beta.-D-glucofuranose	-	-	1.58×10^{7}	6.98	1.63×10^{7}	6.86	1.50×10^{7}	6.03
Esters	Bis(2-ethylhexyl) phthalate	4.17×10^{4}	1.49	5.62×10^{5}	0.25	-	-	-	-
N-compounds	2-Propenamide, N-(aminocarbonyl)-	-	-	2.08×10^{5}	0.09	3.39×10^{5}	0.14	2.91×10^{5}	0.12
	Oxazolidine, 2,2-diethyl-3-methyl-	-	-	1.82×10^{5}	0.08	3.17×10^{5}	0.13	-	-
	Erucamide	5.86×10^{5}	20.99	1.15×10^{6}	0.51	3.07×10^{6}	1.29	1.65×10^{6}	0.66
